# A scoping review of workplace violence against healthcare workers in Singapore

**DOI:** 10.3389/fpubh.2025.1624191

**Published:** 2025-09-16

**Authors:** Phoebe Sing, Arthur Chin Haeng Lau

**Affiliations:** ^1^Yong Loo Lin School of Medicine, National University of Singapore, Singapore, Singapore; ^2^Department of Anatomy, Yong Loo Lin School of Medicine, National University of Singapore, Singapore, Singapore

**Keywords:** workplace abuse, workplace violence (WPV), healthcare worker (HCW), abuse, violence

## Abstract

**Introduction:**

Workplace violence (WPV) has plagued healthcare settings, endangering healthcare workers striving to prioritise patient care. This scoping review aims to explore the prevalence, characteristics, risk factors, and interventions addressing WPV in Singapore’s healthcare sector.

**Methods:**

This scoping review employed the Arksey and O’Malley framework and the PRISMA guidelines. Systematic searches were conducted using MEDLINE, PubMed, Google Scholar, and ScienceDirect for studies published between 2003 and 2024. Grey literature and government reports were also reviewed. The inclusion criteria focused on primary studies conducted in Singapore involving healthcare workers (HCWs) as WPV victims. Data were extracted on study characteristics, prevalence, risk factors, and interventions.

**Results:**

Eight studies met the inclusion criteria, indicating a high and increasing prevalence of WPV. Verbal abuse was the most frequently reported form of abuse, followed by physical violence. Key risk factors included alcohol intoxication and patient dissatisfaction. Existing interventions, such as online reporting systems, self-defence training, and aggression management workshops, lacked standardisation and effectiveness. HCWs have proposed enhanced police protection, advanced alert systems for repeat offenders, stricter legal consequences, and public awareness campaigns.

**Discussion:**

Under-reporting was identified to be a key factor in the persistent prevalence of WPV. The implementation of the Tripartite Framework, along with the development of a robust reporting system, could reduce instances of under-reporting, thereby providing a more accurate representation of the extent of WPV. Employing the Haddon Matrix may offer a comprehensive approach to analysing the issue and informing targeted interventions.

**Conclusion:**

WPV persists in Singapore’s healthcare sector. While the Tripartite Framework is a step forward, further integration of HCW-recommended strategies is required. Future research should evaluate its impact on WPV reduction.

## 1 Introduction

Workplace violence (WPV) has plagued healthcare settings, endangering healthcare workers who strive to prioritise patient care. According to the World Health Organisation, workplace violence can be defined as threats and assaults among healthcare workers (HCWs), which include physical, sexual, verbal, and psychological abuse and workplace harassment (1). It is acknowledged that workplace violence exists in healthcare settings globally (2, 3) and is described as a widespread epidemic affecting all nations (4).

WPV affects HCWs’ physical and mental wellbeing (2) and decreases workplace satisfaction, which leads to reduced professional commitment, ultimately affecting the quality of care and increasing turnover intention (5). These situations are costly and cause significant harm to the healthcare system in the long term.

Efforts have been made to mitigate and curb workplace violence, but despite this, it remains prevalent. In order to prevent WPV and its negative effects, there have been efforts to reduce it through various interventions. For example, in the United States of America (USA), a bill was passed to mandate the Occupational Safety and Health Administration (OSHA) to create a standardised workplace violence prevention plan (6). OSHA’s guidelines include environmental interventions such as panic buttons, door locks, brighter lighting, and accessible exits (7). However, in 2018, HCWs accounted for 78% of all non-fatal workplace injuries and illnesses caused by violence in the USA (8).

Similarly, in Singapore, frequent exposure to workplace violence has resulted in job dissatisfaction, resentment towards patients, and burnout (9). This has led to an increase in the resignation of HCWs, which has reduced the number of available HCWs to provide supporting care (10). This can lead to a negative effect, especially in Singapore’s ageing population (11). Hence, to ensure the quality of care and retention of HCW, it is paramount to implement efforts to reduce the WPV.

As such, individual hospitals in Singapore have implemented protocols to address prevalent and pressing issues. National University Hospital (NUH) has set up online reporting of abuse cases, Alexandra Hospital has assembled the Management of Violence Task Force, and SingHealth has organised workshops on how to manage aggression and violence (12). However, these efforts have not translated into a significant decrease in workplace violence. An article published in 2013 in the national newspaper, The Straits Times, writes about an increasing trend in the number of abusive patients or their next-of-kin. Compared with the current situation in 2023, in which more than 60% of HCWs have seen or encountered abuse in the past year (13), current interventions seem to have little effect, with WPV still being commonplace in the hospital setting.

The Singapore Ministry of Health has acknowledged this issue and has made efforts to reduce abuse. As part of these efforts, the Tripartite Workgroup for the Prevention of Abuse and Harassment of Healthcare Workers was established to implement a zero-tolerance policy to curb workplace abuse. The guidelines were formalised as of 13 December 2023. The framework standardised the definition of WPV, formalised abuse protocols across all institutions, and provided follow-up support for HCWs who have experienced WPV (14).

Despite workplace abuse and the existing policies, there is a paucity of research literature in Singapore to date that discusses the risk factors that can cause such incidents and the different interventions to reduce them. This is evidenced in existing review studies exploring WPV on a global scale in healthcare settings; however, little is known about Singapore. For example, two review studies explored WPV to HCW; although one study by Liu et al. (3) mentioned Asia as a region, there is no mention of Singapore specifically. Meanwhile, another study by Recla-Vamenta et al. (15) mentioned Singapore; however, there were no studies on Singapore included. With limited awareness of the magnitude of workplace violence against HCWs, systemically addressing this issue remains a challenge.

The aim of this scoping review is to explore the current state of workplace violence in the healthcare setting of Singapore, which includes the risk factors, prevalence, characteristics, and interventions. This review can additionally serve to support the initiative by providing insight into the trends of workplace violence as well as suggestions. The research question is, “*What are the current trends of workplace violence in Singapore’s healthcare system?*”

## 2 Methods

This scoping review employs the framework proposed by Arksey and O’Malley (16) and adheres to the PRISMA reporting standards for scoping reviews (17). The review proceeded through five distinct stages: (1) identifying the research question, (2) identifying relevant studies, (3) study selection, (4) charting the data, and (5) collating, summarising, and reporting the results.

### 2.1 Identifying relevant studies

A literature search was conducted on MEDLINE, PubMed, Google Scholar, and ScienceDirect for papers published in the past 20 years, from 2003 to 2024. The search string consisted of the keywords (“healthcare” OR “frontline” OR “medical” OR “hospital”) AND (“abuse” OR “violence” OR “aggressions” OR “mistreatment” OR “incivility”) AND (“Singapore”). Additionally, reference lists of included studies and reviews on similar topics were hand-searched from the grey literature, which included news articles, search engines, and government websites, for further relevant studies. The word “healthcare” was used instead of “healthcare professional” as it is a more generic term that yielded relevant search results.

### 2.2 Study selection

All identified articles were initially screened based on the title and abstract, and eligibility was assessed based on the full text. To answer the research question, the inclusion criteria were as follows.

(1) Published in the last 21 years, from 2003 to 2024.
(2) Located in Singapore.
(3) Involves abuse by the patient or the patient’s family members towards the healthcare worker.
(4) Primary study.

Any disagreement during the evaluation of the eligibility of the articles will be discussed until a consensus is reached. To calculate inter-rater reliability, both the percentage agreement and the Kappa coefficient were determined.

In the context of this review, HCWs are defined as the staff of healthcare institutions, including doctors, nurses, and emergency medical services. We acknowledge that healthcare workers can also perpetrate abuse. However, to support the Tripartite Workgroup for the Prevention of Abuse and Harassment of Healthcare Workers, we decided to focus on abuse from patients towards HCWs. The last search date was 5 June 2025, after which it was agreed that no additional studies would be included in the analysis.

### 2.3 Data extraction

Eight studies were included in this review. The following information was charted onto a data extraction sheet:

1. Research title, author, year of publication
2. Type of study
3. Aim of the study
4. Study populations
5. Variable measured
6. Important results

## 3 Results

**Figure 1:**
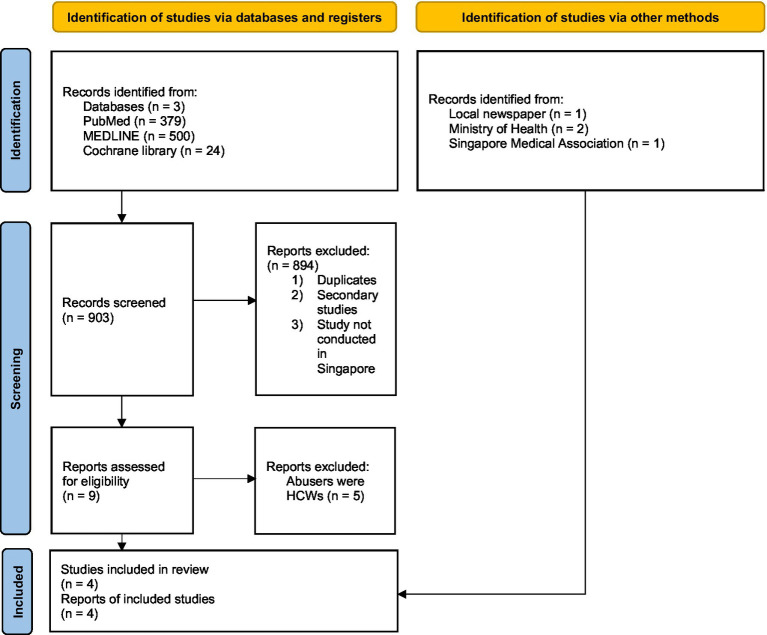
Flow diagram of the literature search.

A total of 379 studies were identified from PubMed, 500 from MEDLINE, and 24 from Cochrane Library, totalling 903 studies. A total of 894 studies were excluded after their abstracts and titles were screened for eligibility, as they were either duplicates, secondary studies, or were not conducted in Singapore. Five studies were excluded after full-text screening because the abusers were HCWs. The remaining four studies were included in the review. Grey literature was searched from local news, such as The Straits Times and the Ministry of Health, and four studies were considered eligible for the review. Eight studies were included in this review. The selection process is shown in Figure 1. There was disagreement between the two articles, which resulted in a percentage agreement of 84.6% and a Kappa coefficient of 0.68. Both were considered very good agreement or a moderate level of agreement, respectively. There were disagreements on two papers, which were discussed, and a consensus was reached. The findings of this review are organised into four sections: prevalence, reasons for abuse, formal interventions to mitigate WPV, and suggested interventions by HCWs to mitigate WPV. The studies included in this review are shown in Table 1.

**Table 1:** Included studies.

Research title	Type of study	Aim of the study	Variable measured	Study population and demographics	Key findings
Self-reported incidence of verbal and physical violence against emergency medical services (EMS) personnel in Singapore (18)	Cross-sectional survey	To investigate the occurrences of self-reported incidences of physical and verbal violence against EMS personnel To explore, from the EMS personnel viewpoint, the possible provoking factors and reasons for not reporting abuse and possible interventions to reduce abuse against EMS	Verbal abuse is defined as the use of profanity and shouting intended to distress and threaten the EMS personnel Physical abuse is defined as hitting, spitting, or using a weapon intended to cause physical injury to the EMS personnel	EMS personnel (*n* = 246) of the Singapore Civil Defence Force They included 144 males (58.5%) and 102 females (41.5%).	The study found that 64% of EMS personnel experienced verbal abuse and 16.3% experienced physical abuse in the past year. Verbal abuse was primarily attributed to alcohol intoxication and dissatisfaction with EMS policies, while physical abuse was linked to alcohol intoxication and patients’ medical conditions, such as post-seizure confusion, stroke, or altered mental status. Although 78% of EMS personnel knew the reporting process and 82.1% were aware of available mental health support, only 50.8% officially filed a report of verbal abuse, and 79.3% officially filed a report of physical abuse. Barriers included empathy for aggressors, minimal injuries, and the complexity of reporting. To reduce abuse, EMS personnel suggested faster police deployment, police protection for high-risk cases, stricter penalties for abusers, and warning systems for identifying previous offenders. Additionally, they recommended self-defence training, soft patient restraints, and body-worn cameras to enhance safety and protection.
Violence in the workplace—A survey on the experience of doctors in Singapore (12)	Cross-sectional survey	To investigate the prevalence and magnitude of abuse towards physicians in healthcare To investigate the effects of such abuse on physicians and the general public	Verbal abuse is defined as the use of profanity, threatening remarks, and harassment	Doctors (*n* = 251) across healthcare institutions in Singapore	The study found that in the past year, 48% of doctors encountered at least one verbal abuse case, while 16% of doctors encountered at least one physical abuse case. To curb abuse, the existing interventions have been employed by Singapore hospitals: National University Hospital: Online reporting system for abuse Alexandra Hospital: Response team for the regulation of abuse, self-defence training, and refusing admittance of very violent individuals SingHealth hospitals: Workshops focused on handling aggression To further reduce abuse, doctors suggested these interventions A campaign utilising posters and caution signs to advocate against violent conduct Develop an established protocol to undertake after abuse Discreet alert systems in vulnerable locations Accompaniment system Structural barricades in the interior design Training of risk appraisal and de-escalation
Nursing management of aggression in a Singapore emergency department: A qualitative study (9)	Qualitative Study	Explore nurses’ perceptions of managing aggressive patients presenting to the Emergency Department.	The focus group interviews were conducted in English, and all were digitally recorded, with each lasting between 30 and 60 min Impact of patients’ aggressive behaviours on nurses (psychologically & physically) Nursing assessment of aggressive behaviours (similar to risk assessment) Nursing management of aggressive behaviours (caring and maintaining professionalism with aggressive patients) Organisational support and responsiveness (learning on the job)	Registered Nurses (*n* = 10) aged 22–30 years, who had prior contact with aggressive patients, were interviewed. The participants had spent their entire nursing careers in the 24-h Emergency Department at a major Singapore general acute hospital and had 2–8 years of working experience.	All interviewees indicated that alcohol intoxication contributed to a higher likelihood of aggressive behaviours, followed by an underlying medical condition such as sepsis, electrolyte imbalances, heat stroke, dementia, and seizures. Existing PREVENTION measures implemented by nurses 1. Nursing assessment Nurses would assess patients for the potential to be aggressive based on their presenting information, previous history, pattern recognition of abusive patients based on past experiences, and medical diagnosis. Medical diagnoses that they will consider potentially aggressive are alcohol intoxication or delirium Patients with a previous history of drug overdose or risk of self-injury will be considered potentially aggressive as well. By paying attention to nonverbal cues, speech patterns, and indications of discomfort or discontent through the patient’s words, tone, and volume, nurses can pattern-recognise a potential aggressor. However, there were no formal guidelines, and hence, a systematic form of assessment to help identify people who can potentially turn aggressive should be provided. It appeared that current guidelines were inadequate, as they focused on when to alert the police and seek assistance. 2. Turnover In cases where nurses were unable to uphold their professionalism with patients, colleagues would take the initiative to step in to take over the duty of care. Regular interaction with patients was considered essential to prevent aggression, which involved offering information and updates. If language barriers were present, hospital translators were utilised to facilitate communication. Existing PROTECTION measures implemented by nurses 1. Equipment Nurses employed chemical and physical restraints to regulate aggressive behaviours to maintain safety, allowing healthcare staff to carry out undisrupted important clinical procedures. If measures are still unable to regulate the aggression, nurses resort to seeking help from the security department and the police. 2. Police If severe physical aggression was detected, nurses would notify the police, and security personnel would promptly arrive to support the nurses during the aggressive incidents. However, nurses hope to be better supported. They hoped the system would punish aggressors on their behalf, rather than them having to take the action to pursue the aggressors. Nurses reported contacting the police if it was perceived to be a case of severe physical aggression or when the person threatened the nurse. Nurses reported that most of the security guards were quick in their arrival and assisted the nurses during aggressive incidents 3. Organisational structure Formal debriefing sessions following abusive incidents were uncommon, and in some cases, none were organised at all. Nursing managers often did not acknowledge incidents deemed insignificant or not serious enough. These events were frequently justified and normalised due to the high frequency of aggressive encounters. The majority of nurses turned to their colleagues for emotional support. They expected frontline leaders, such as senior nurses, to possess the skills to manage aggressive situations and serve as role models. However, these leaders did not meet those expectations. 4. Formal guidelines Not all nurses reported being familiar with the available guidelines, policies, and procedures that could help them manage aggressive incidents. It was suggested that the guidelines should provide a systematic form of assessment to identify people who can potentially turn aggressive. However, current guidelines, protocols, and policies provide directions on when to call the police and seek help. It appears that most nurses filed police reports when they received verbal threats from their patients: It was compulsory for nurses to write incident reports for physical abuse as part of protocol. But in the case of verbal abuse, the decision to file a report was left to their discretion. They believed that every form of abuse should be documented in an incident report to protect against potential legal liabilities in the event of allegations of negligence or complaints against the nurse. 5. Reporting Although incident reporting was considered important, the process was arduous and deemed futile, as management rarely took action on the reports or provided additional resources to help nurses handle aggressive incidents. 6. Education Workplace education, preparation, and training were considered crucial to equip nurses in the emergency department for handling aggressive behaviours. Some nurses emphasised that real-world experience was key to developing the necessary skills, knowledge, and confidence. They recommended incorporating aggression-management training programmes to boost their confidence, alongside strengthening existing induction programmes. Currently, they primarily learn from more experienced colleagues. 7. Coping Nurses advocated for the creation of resources to help them manage workplace challenges, and they highly valued security and police as key resources for handling aggression.
Exposure to crises and resiliency of healthcare workers in Singapore (35)	Mixed Method	Examine exposure and emotional resiliency to crises, such as aggression or violence from patients and relatives, of healthcare workers in Singapore.	Questionnaires asked about Mental health training Exposure to work-related crises (such as aggression and violence from patients and/or relatives) Personal crises (relationship conflicts, sudden/unexpected death of a loved one, financial or health problems).	Healthcare workers (*n* = 496) in seven public hospitals in Singapore. 81% were female, 50% were nurses, and 35% had mental health training.	The study found that more than 70% of hospital staff experienced workplace violence. After experiencing workplace violence, it was found that with mental health training, HCWs were more resilient after the encounter. It enhanced their confidence, making them better equipped to handle unexpected situations and more likely to approach challenges with a calm, composed attitude. Those who had received mental health training were twice as likely to demonstrate greater resilience compared to those who had not undergone such training, proving mental health training valuable as a post-abuse intervention. However, less than half of HCWs were considered resilient.
Findings and recommendations of the tripartite workgroup for the prevention of abuse and harassment of healthcare workers (13)	News article	Not applicable	Not applicable	Not applicable	The study found that more than two-thirds of the 3,000 HCWs surveyed by the Tripartite Workgroup for the Prevention of Abuse and Harassment of Healthcare Workers in 2022 had seen or had a firsthand encounter of abuse and harassment. To curb WPV, the government has devised a triple P, Promote, Prevent, Protect, approach. Under promote, the government hopes to promote constructive relationships between healthcare workers and patients through launching a National Public Education Campaign to clarify the responsibilities of healthcare workers’ roles and foster respect for them. Under prevention, the government hopes to prevent incidents of abuse and harassment by equipping healthcare workers with the skills to manage and avoid potentially abusive situations, while deterring individuals who may engage in abusive behaviour through a strict zero-tolerance policy. Through a strict zero-tolerance policy. Under the protection, the government hopes to protect healthcare workers who experience abuse and harassment by establishing a zero-tolerance policy accompanied by comprehensive protocols for addressing such incidents. This should include: A standardised definition of abuse and harassment to ensure consistency in understanding what constitutes abusive behaviour. A well-defined protocol for reporting and escalating incidents A supportive reporting culture that encourages individuals to come forward. Well-defined and consistently enforced consequences for those involved in abusive conduct
Abuse of Healthcare Workers (39)	Opinion Article	Not applicable	Not applicable	Not applicable	The study found that reported cases of abuse towards HCW doubled in a short span of 2 years, from 16 in 2010 to 33 in 2012. Further, up to 70% of HCWs in Singapore have experienced physical abuse, with primary perpetrators being older male patients who have neuropsychiatric disorders or are under the influence of drugs or alcohol.
More healthcare workers are facing abuse (19)	News article	Not applicable	Not applicable	Not applicable	The study found that to curb WPV, HCWs at TTSH have been taught escalation protocols when encountering abusive patients. There are also emergency alert buttons at each ward, which HCWs can use to signal if they feel at risk or in danger. When activated, colleagues from adjacent wards and security staff will be notified and provide assistance where they can. Across all HCWs, there were 1,080 abuse cases reported in 2018, 1,200 cases in 2019, and 1,300 cases in 2020. At TTSH, there were 218 reported abuse cases in 2019, 158 cases in 2020, and 244 in the first 10 months of 2021. At Singapore General Hospital (SGH), there were 70 reported abuse cases in 2017, 170 cases in 2020, and 180 cases in the first 10 months of 2021. At Ng Teng Fong General Hospital, there were 38 reported abuse cases every year from 2017 to 2020 and 35 cases in the first 10 months of 2021. At Khoo Teck Puat Hospital, reported abuse cases almost doubled from 2016 to 2020.
Workplace Harassment in Singapore’s Healthcare Sector (40)	Government presentation	Not applicable	Not applicable	Not applicable	In November 2014, the government introduced an enhancement to the Protection from Harassment Act (POHA), offering additional safeguards for public healthcare workers who provide essential services. Under this legislation, offenders may face a fine of up to $5,000, imprisonment for up to 6 months, or both. Victims of harassment can seek protection orders to restrain perpetrators or pursue legal action for damages. The penalties are more severe when the offence is committed against public sector workers during the course of their duties. Additionally, certain private sector healthcare workers, such as those providing outsourced paramedic services to the Singapore Civil Defence Force (SCDF), are also granted enhanced protection due to their role in delivering public services. The study found that between 2018 and 2020, under the POHA, the number of cases reported to the police rose from 40 to 58. However, this may not fully reflect the actual incidence of such cases, as healthcare workers may opt to exercise empathy and refrain from taking legal action or escalating every altercation. To further support the mental health of healthcare workers, various initiatives have been implemented, including the provision of counselling hotlines, the establishment of wellbeing offices within healthcare clusters to address staff mental health concerns, and the introduction of digital solutions such as the WYSA app, which offers 24/7 mental health support.

### 3.1 Prevalence of abuse towards HCW

Data have shown that the abuse of HCWs is high and is increasing in Singapore. In 2009, the Singapore Medical Association found that 48% (*n* = 120) of doctors encountered 1–3 verbal abuse and harassment cases, including swearing, intimidation, and verbal threats. Of the 120 doctors, 16 doctors had experienced an additional 1–3 physical abuse cases involving slapping, punching, kicking, and biting over the same time period. The remaining 52% (*n* = 131) of doctors were unharmed and did not encounter any abuse (12).

Compared with other studies, this is also an occurrence targeted towards other HCWs. A study by Tay et al. (18) surveyed 246 paramedics from the Singapore Civil Defence Force over a period of 1 year and found that 64% (*n* = 159) of paramedics experienced at least one verbal abuse with harassment which included shouting and use of offensive language with the intent of intimidating the paramedic, and 16.3% (*n* = 40) of the paramedics also experienced at least one physical abuse and harassment, including punching, slapping, kicking, and spitting with the intent of causing physical harm to the paramedics (18).

In the wider population of Singapore’s HCWs, more than two-thirds of the 3,000 HCWs surveyed by the Tripartite Workgroup for the Prevention of Abuse and Harassment of Healthcare Workers in 2022 had seen or had their first encounter of abuse and harassment (13).

An additional finding is that while there are high occurrence rates of WPV, they are also increasing. According to a news article by Tan Tock Seng Hospital in 2021, abuse and harassment cases at Singapore’s public healthcare institutions have been steadily increasing over the years. 1,080 cases were reported in 2018, 1,200 cases in 2019, and 1,300 cases in 2020 (19).

Individual hospitals followed a similar trend. Tan Tock Seng Hospital reported 218 cases in 2019 and 244 cases in the first 10 months of 2021. Singapore General Hospital reported 70 cases in 2017, 170 cases in 2020, and 180 cases in the first 10 months of 2021 (19). These data are presented in Table 2, which shows the average number of cases per month. The data were then converted to show the prevalence rates using data from Singapore Registered Health Personnel (41) which is available online (Table 3).

**Table 2:** Abuse data towards HCWs in Singapore as reported by the Singapore government.

Institution	Average cases of workplace abuse and harassment to healthcare workers per month as reported by the Singaporean government			
	2018	2019	2020	2021
Singapore General Hospital	X	X	14	18
Ng Teng Fong General Hospital	3	3	3	4
Changi General Hospital	X	X	17	11
Tan Tock Seng General Hospital	X	18	13	24

*KTP cases almost doubled from 2016 to 2020. *All public healthcare includes SGH, NTFGH, CGH, TTSH. *No data obtained for other public healthcare institutions as of yet.

**Table 3:** Prevalence rate of abuse towards HCWs in Singapore as reported by the Singapore government.

Metric	Year		
	2018	2019	2020
No. of total cases	1,080	1,200	1,300
Total no. of HCWs	33,575	34,561	36,399
Prevalence Rate (%)	3.21	3.47	3.57

### 3.2 Reasons for abuse towards HCW

It is paramount to identify the reasons for abuse, as these are potential risk factors that HCWs can look for and be more vigilant against. A study by Tay et al. in 2020 (18) collated different reasons for abuse and their proportions. Alcohol intoxication levels of patients and patient dissatisfaction with EMS policies were found to be potential reasons for the verbal abuse of HCWs. Patient dissatisfaction resulted in 25.8% (*n* = 64) of abuse cases, and alcohol intoxication contributed to 52.0% (*n* = 128) of abuse cases. This makes alcohol intoxication the biggest contributing factor and a high-risk factor (18).

In the same study conducted by Tay et al. (18), the majority of the respondents (62.6%, *n* = 154) found that alcohol intoxication was the main reason for physical abuse incidents. The second reason was medical conditions; the study found that 27.6% (*n* = 68) of the patients had post-seizure confusion, stroke, and altered mental status. Additionally, it was further observed that 67.5% (*n* = 166) of these cases occurred in public, and 85% (*n* = 209) of physical assailants were male (18). This study suggests that the contributing factor to both forms of abuse of HCWs is alcohol influence. Following that, patient dissatisfaction often culminates in verbal abuse, whereas medical conditions result in physical abuse.

Similarly, another study conducted by Tan et al. in 2015 (9) found that one of the major indications for potential abuse was alcohol intoxication, followed by underlying medical conditions such as sepsis and dementia. These indications imply that alcohol intoxication followed by medical conditions is the biggest risk factor for abuse to HCW (9). Both studies corroborate that alcohol influence is the biggest reason for physical and verbal abuse in HCWs and that medical conditions are the second biggest reason for physical abuse. A study by the MOH in 2022 also found other root causes of abuse towards HCWs, such as racial discrimination, mismatched expectations of HCW’s roles, and limited manpower (13).

### 3.3 Formal interventions

In the context of current collective interventions to curb abuse of healthcare workers, this remains scarce in the current literature. A few studies state the current measures implemented by individual hospitals in Singapore. National University Hospital has an accessible online platform through which HCWs can report abuse cases. Alexandra Hospital has a Management of Violence Taskforce and sends its new nurses to self-defence classes. SingHealth hospitals, such as Tan Tock Seng Hospital, have workshops on managing aggression and violence. The Institute of Mental Health put up deterrence posters that warn patients of the consequences of abuse towards HCWs, installed physical blockades, and sent their HCWs for risk management training (12). The interventions implemented in individual hospitals are summarised in Table 4.

**Table 4:** Interventions to curb WPVs against HCWs in individual hospitals in Singapore.

Hospital	Protect			Prevent	Promote
	Definition of abuse & harassment	Standardisation of protocols	Follow-up actions	Equip HCWs	Public education campaign
National University Hospital	NIL	NIL	✓	NIL	NIL
Alexandra Hospital	NIL	✓	NIL	✓	NIL
SingHealth Hospitals	NIL	NIL	NIL	✓	NIL

*NIL is indicated when no data is available.

By contrasting current hospital interventions with government interventions, it suggests that existing hospital interventions are minimal and warrant a shift towards current government interventions.

### 3.4 Suggested interventions by HCW

Tay et al. (18) and Tan et al. (9) also show interventions suggested by HCWs, which suggested the following:

1) Police protection:

Swift police response timing when encountering violent patients. For example, alcohol intoxication or a dispute. Further, an advanced warning system should be included to initiate police protection for former offenders of violence.

2) Substantial repercussions:

Offenders of violence against HCWs deserved more serious punishment than the lacklustre ones, and there was a need to raise public awareness of the issue through campaigns.

3) Advanced alert system:

Individuals with a history of perpetrating violence against HCWs should be flagged through an advanced alert system in order to initiate appropriate security measures for their encounters with HCWs.

4) Self-defence training:

Self-defence training would help HCWs become more equipped to protect themselves in the event of physical abuse.

5) Equipment:

HCWs should be trained in using various equipment, such as patient restraints and body-worn cameras, as these help to reduce the risk of abuse of HCWs.

6) Raising Public Awareness:

Raising public awareness of this issue will reduce workplace violence towards HCWs.

7) Organisational structure:

Front-line leaders, such as senior HCWs, possess the competency to handle aggressive incidents and serve as their role models. Furthermore, HCWs found that management rarely took action on reports or provided resources to support HCWs in handling aggressive incidents.

8) Education:

Training programmes, such as programmes for aggression management, will help better equip the HCWs.

9) Environment:

The installation of silent alarm systems, warning signs at the front counters, and poster campaigns will help deter potential aggressors from causing abuse.

## 4 Discussion

This scoping review provides an extensive exploration of WPV against HCWs in Singapore, underscoring the complex interplay between prevalence, contributing factors, existing interventions, and recommendations for improvement. Despite increasing attention, WPV remains prevalent and appears to be escalating, driven primarily by factors such as alcohol intoxication and patient dissatisfaction. Effective WPV management requires a nuanced understanding of under-reporting mechanisms, targeted preventive measures, and comprehensive frameworks to guide interventions.

### 4.1 Under-reporting

Although not mentioned in the literature, a possible reason for the prevalence of abuse is the culture of under-reporting.

For example, a study by Arnetz et al. (20) in 2015 identified under-reporting of workplace violence as a critical barrier to reducing the occurrence of workplace violence. Under-reporting obscures the true extent of the issue, preventing the formulation of effective injury prevention strategies, thereby sustaining the prevalence of abuse.

This is similarly seen in other countries as well, such as Australia, Sweden, the United States, Canada, and China (21–25).

In Singapore, 50% of HCWs would report a case of abuse even though 78% of them knew the steps to make an abuse report (18).

The reasons for under-reporting can be empathy for perpetrators (45.1%), harm inflicted was not severe enough (62.2%), and the cumbersome reporting process (68.3%) (18). Therefore, the reasons for under-reporting can be divided into different categories: individual, interpersonal, and systemic reasons.

#### 4.1.1 Individual reasons

An individual’s reasoning for under-reporting could have stemmed from HCW normalising or rationalising acts of abuse as not being abuse. A potential reason why acts of abuse are being normalised could be that the abuse is not significant or severe enough to be perceived as abuse. As a result, acts of abuse have become normalised and have not been reported. Further, a significant number of healthcare workers had rationalised abuse as part of the job, normalising it. Without a clear definition or understanding of abuse, such acts are under-reported (13).

#### 4.1.2 Interpersonal reasons

Nurses often face ethical dilemmas when dealing with violence in the hands of a patient, making it more difficult for the covenant of the nurse–patient relationship. A product of the nurse–patient relationship is the building of rapport; in the event of abuse, nurses may feel empathy towards the patient (18). As a result, this may make them less inclined to file a report against their perpetrators and their patients themselves in order to protect them from possible consequences.

#### 4.1.3 Systemic reasons

Further, under-reporting may be due to the lack of confidence in the system at punishing the aggressor, fuelled by management’s personal experience that rarely acts on reports or provides additional resources to support HCWs in managing aggressive incidents (9). This can be explained by the helpless trial framework proposed by Krishna et al. in 2023 (26). The framework suggests that victims of workplace aggression end up with an acquired state of helplessness. It involves six elements to create a learned state of helplessness: encounter, assessment of the situation, reaction, a futile response, helplessness, and withdrawal into a more permanent helpless state of mind. The elements of an encounter, a reaction, and a futile response in which superiors did not act on reports can be observed in the nurses’ experience cited in the study of Tan et al. This eventually leads to a lack of confidence in the organisation, which can be seen as similar to a withdrawal into a more permanent helpless state of mind and may eventually breed a culture of under-reporting (26). Helpless trials highlighted the reasons for workplace aggression. Conversely, helpless trials also highlight that successful intervention, in which a victim sees an adequate response to their encounter, can break the cycle to de-escalate workplace bullying.

### 4.2 Solving under-reporting

Under-reporting can hinder violence prevention measures by underestimating the true extent of an issue. This underestimation creates a misleading perception that less preventive action is necessary than required, thereby potentially limiting the effectiveness of prevention efforts (27).

In the absence of comprehensive knowledge about the full range of violent events, preventive efforts are only formulated for a limited set of issues (20). Therefore, it is imperative to address under-reporting to mitigate workplace violence.

In December 2023, the Tripartite Workgroup released a framework for the prevention of abuse and harassment in healthcare. The document details a newly implemented zero-tolerance policy against workplace violence in healthcare and includes a protocol for incident response, reporting, post-incident management, and a common definition of abuse. By viewing workplace violence as intolerable, HCWs would report incidents of abuse and solve under-reporting problems.

A zero-tolerance policy must be paired with an efficient reporting system. The cumbersome reporting process is one of the major reasons cited for under-reporting (18). In addition, there were increased instances of burnout. This was due to the fact that if reporting systems are time-consuming, a heightened focus on incident reporting may intensify burnout by increasing the overall workload (28).

### 4.3 Solving workplace violence

Curbing the culture of under-reporting can support the unveiling of an accurate magnitude of workplace violence, allowing the means of solving workplace violence through the formulation of prevention by frontline HCWs who possess direct experience and insight into the challenges at hand. They have raised the need for police protection, substantial repercussions, and a supportive organisational structure, which is now echoed by the tripartite workgroup at the governmental level. The multitude of suggested interventions by HCWs suggests that hospitals in Singapore should take more time to overcome the abuse of HCWs. The Tripartite Workgroup offered suggestions to mitigate workplace violence, known as the 3 Ps (13): ‘*Promote*’ better HCW-community relationships, ‘*Protection*’ of HCWs through a zero-tolerance policy with effective systemic protocols, and ‘*Prevention*’ of abuse and harassment by equipping healthcare workers (Table 5).

**Table 5:** Tripartite framework for the prevention of abuse and harassment of HCWs.

Protect	Prevent	Promote
Protect healthcare workers who face abuse and harassment	Prevent situations that lead to abuse and harassment	Promote positive relationships between healthcare workers and patients/caregivers
Develop a zero-tolerance policy with effective protocols for handling abuse and harassment. This includes: A common definition of abuse and harrassment An effective reporting and escalation protocol A supportive culture of reporting Clear consequences that are implemented and enforced	Equip healthcare workers to avoid potential abusive situations Deter potential offenders with the zero-tolerance policy	Align expectations of healthcare workers’ roles and promote respect towards them
Standardised zero-tolerance policy across institutions		National public education campaign

By aligning healthcare workers’ (HCWs) recommendations with forthcoming 3P government initiatives, this convergence suggests that government policies address HCWs’ needs. For example, studies suggested measures to protect healthcare workers through the application of organisational structures, quicker police response, and more severe sentences (9, 18), which are included in government initiatives under ‘*Protect’* and ‘*Prevent’*. Hence, by directly addressing the specific needs of HCWs, government interventions have the potential to significantly reduce instances of abuse, fostering a secure environment that enables them to deliver high-quality care.

Conversely, HCW recommendations are not mentioned in the government-recommended interventions. For example, the flagging system, equipment, and environmental components could help deter abuse towards them. Such interventions can be taken into consideration for future implementation, as previous studies have found that environmental interventions, such as alarm systems and patient restraints, account for the majority of mitigating factors affecting WPV (29–31).

Government interventions also cite the ‘*promotion*’ of positive relations between HCWs and patients or caregivers, which is not raised among the many HCW recommendations but still has an impact on HCWs. By establishing coherent roles and expectations of HCWs and promoting respect towards HCWs, positive relationships between HCWs and patients or caregivers can be fostered. Such a correlation is accurate, as patient satisfaction depends on whether patient expectations are met (32). Hence, by establishing the roles and expectations of an HCW, patients know what to expect of HCWs, and HCWs who do their jobs meet patients’ realistic and accurate expectations, thus improving their satisfaction. Since patient dissatisfaction is the second biggest cause of verbal abuse towards HCWs, reducing patient dissatisfaction can reduce verbal abuse towards HCWs and improve patient relations between HCWs and patients or caregivers.

It is also recognised that the Haddon Matrix offers a different lens from the 3Ps (33). The Haddon matrix, originally developed and applied for road safety prevention, can be adapted for occupational health as a measure to prevent injuries. The Haddon matrix integrates the host, vector, vehicle, and environmental factors to develop strategies at the primary, secondary, and tertiary levels. When applying the Haddon matrix to the prevention of workplace violence in a healthcare setting, the host refers to healthcare workers who are vulnerable to abuse. The vehicle represents the object that can cause abuse (such as a profanity in verbal abuse and a chair in physical abuse), while the vector or agent is the patient or their family member who causes abuse. The environment encompasses both the physical setting (such as the ward) and social context (such as the organisational structure and its policies). Additionally, these categories are further divided into stages: before, during, and after an incident of abuse (33). The proposed strategies to mitigate workplace violence by integrating the 3Ps into the Haddon Matrix are presented in Table 6.

**Table 6:** Suggestions by HCWs and 3Ps adapted into Haddon matrix.

Abuse phase	Host factors (Individual)	Vector and vehicle factors (Interpersonal)	Physical/social environmental factors (Systemic)
Before abuse	Prevent Denial of entry to violent individuals Educate healthcare workers to avoid and de-escalate potential abusive situations Patient risk assessment of abuse	Promote Ensure coherent expectations of healthcare workers’ roles to encourage appreciation for them through a nationwide campaign	Protect Police Protection Advanced alert system Deter potential offenders by enforcing consequences against workplace violence Organisational structure that enforces the zero-tolerance policy
During abuse	Protect Self-Defence training Management of aggression and violence Efficient police response deployment		Protect Equipment such as patient restraints and body-worn cameras Environmental elements such as a panic button and accessible exits
After abuse	Coping Mental health resources		Reporting Voluntary online reporting of abuse cases Substantial repercussions for aggressors

The Haddon Matrix provides a better understanding of how to mitigate abuse. This highlights the need to break down the problem of workplace violence into smaller and more manageable components. This is followed by targeting every stage and element of abuse. For example, by considering the three factors before an abuse case, under host factors, individual HCWs must be trained to identify patients who have a higher potential of abusing HCWs through risk assessment and avoid or de-escalate any potential abuse situation. Under vector and vehicle factors, good interpersonal relationships between HCWs and the wider community should be promoted by ensuring coherent expectations of HCWs through a national campaign. Finally, under environmental factors, institutions need to ensure that the relevant security forces are conducting proper patrols to ensure an adequate level of police protection.

Interventions by the tripartite workgroup also ensure the recovery of staff who are victims of workplace abuse by providing ‘mental health support’ (14). However, ‘mental health support’ may lack specificity in terms of what strategies the tripartite framework is directed toward. One suggestion to support HCWs’ mental health is to strengthen coping methods and build resilience. Coping is defined as “*thoughts and behaviours that people use to manage the internal and external demands of situations that are appraised as stressful*” (34). It provides the individual with closure after abuse has occurred. Some examples of coping methods include mental health training, problem-solving skills, social support, and material resources. Mental health training, such as mental health-related talks and certified therapy programmes, helped HCWs handle unexpected events such as workplace violence more efficiently, making them more resilient (35). Resilience, defined as a change in the way adversity is appraised (36), has long-term benefits that are not limited to the incident. Both coping and resilience come hand-in-hand, and applying a range of effective coping methods can help HCWs to achieve resilience (37).

The long-term adaptation outcomes of coping strategies and resilience building are beneficial for HCWs in maintaining the quality of Singapore’s healthcare. Therefore, mental health interventions such as strengthening coping methods and resilience building should be specified in the integrated Haddon Matrix, under the stage of ‘After abuse’.

The above are suggestions for solving WPV, which were adapted from the Haddon matrix, 3Ps, and suggestions by the HCW. However, despite these suggestions, few have been implemented in hospitals (9). This suggests the need to implement strategies to curb WPV in hospitals to protect HCWs. On the other hand, given the recent introduction of the tripartite framework in December 2023, it may be possible that these strategies are currently being applied to curb WPV. However, owing to the scarcity of current data on WPV, further studies should be carried out to investigate the impact of the tripartite framework.

The study contributes by providing a framework for understanding WPV, solving under-reporting, and solving WPV through the integration of the 3Ps into the Haddon Matrix. Although this is contextualised to SG, it can be transferable to support curbing WPV in other countries. A review by Spelten et al. in 2020 (38) also adapted the Haddon Matrix to reduce WPV, highlighting the importance of interventions at all stages of violence to reduce WPV. However, as this study was conducted in Western countries and contextualised in psychiatric wards and nursing home settings, it may not be representative of an entire healthcare institution.

### 4.4 Limitation

This scoping review has several limitations. First, the study is constrained by the scarcity of research specifically addressing WPV in Singapore’s healthcare sector, limiting the depth of analysis and generalisability of the findings. Second, this review study can be limited by the under-reporting of the published literature on WPV. As discussed above, WPV tends to be under-reported; it is possible that the published literature, which only documents WPV, may be underestimated. Third, although this review study has contributed to curbing WPV based on the Tripartite Framework for the Prevention of Abuse and Harassment of Healthcare Workers, it should be taken with caution due to the lack of evidence of its effectiveness in curbing workplace violence, as it was only introduced in 2023. Future studies should assess the long-term effectiveness of recent interventions and explore broader workplace dynamics that contribute to WPV.

## 5 Conclusion

This scoping review highlights the persistent and growing issue of workplace violence (WPV) in Singapore’s healthcare sector, exacerbated by under-reporting and systemic challenges. The findings underscore the critical need for comprehensive interventions, including standardised reporting protocols, stronger legal repercussions, and enhanced protective systemic measures for healthcare workers. While the newly introduced Tripartite Framework marks a significant step forward, gaps remain in its implementation, particularly in addressing environmental deterrents and frontline worker resilience. Integrating the Haddon Matrix with its three perspectives of individual, interpersonal, and systemic factors, and further into the three phases of pre-abuse, during abuse, and post-abuse, the Tripartite Framework could offer a more holistic approach to WPV prevention, subduing the incident from start to end. Future research should evaluate the effectiveness of the framework and explore additional strategies to foster a safer and more sustainable working environment in Singapore’s healthcare sector.

## Data Availability

The original contributions presented in the study are included in the article/supplementary material; further inquiries can be directed to the corresponding author.

## References

[1] World Health Organization. Violence and harassment. (2024). Available online at: https://www.who.int/tools/occupational-hazards-in-health-sector/violence-harassment (Accessed February 7, 2024).

[2] Caruso R Toffanin T Folesani F Biancosino B Romagnolo F Riba MB . Violence against physicians in the workplace: trends, causes, consequences, and strategies for intervention. Curr Psychiatry Rep. (2022) 24:911–24. doi: 10.1007/s11920-022-01398-1, PMID: 36445636 PMC9707179

[3] Liu J Gan Y Jiang H Li L Dwyer R Lu K . Prevalence of workplace violence against healthcare workers: a systematic review and meta-analysis. Occup Environ Med. (2019) 76:927–37. doi: 10.1136/oemed-2019-105849, PMID: 31611310

[4] Kingma M. Workplace violence in the health sector: a problem of epidemic proportion. Int Nurs Rev. (2001) 48:129–30. doi: 10.1046/j.1466-7657.2001.00094.x, PMID: 11558684

[5] Pariona-Cabrera P Cavanagh J Bartram T. Workplace violence against nurses in healthcare and the role of human resource management: a systematic review of the literature. J Adv Nurs. (2020) 76:1581–93. doi: 10.1111/jan.14352, PMID: 32175613

[6] National Nurses United. Members of Congress introduce bill to prevent violence in health care, social service workplaces|National Nurses United. (2023). Available online at: https://www.nationalnursesunited.org/press/members-of-congress-introduce-bill-to-prevent-violence-in-health-care-and-social-service-workplaces (Acceseed October 11, 2023).

[7] Occupational Safety and Health Act of 1970. Guidelines for Preventing Workplace Violence for Healthcare and Social Service Workers. Occupational Safety and Health Act of 1970. (2016). Available online at: https://www.osha.gov/sites/default/files/publications/osha3148.pdf (Acceseed October 11, 2023).

[8] U.S. Bureau of Labor Statistics. Workplace violence in healthcare. (2020). Available online at: https://www.bls.gov/iif/factsheets/workplace-violence-healthcare-2018.htm (Acceseed October 11, 2023).

[9] Tan MF Lopez V Cleary M. Nursing management of aggression in a Singapore emergency department: a qualitative study. Nurs Health Sci. (2015) 17:307–12. doi: 10.1111/nhs.12188, PMID: 26031693

[10] Yip C Chia L. Why some healthcare workers in Singapore’s hospitals have quit—and others soldier on. CNA. (2022). Available online at: https://www.channelnewsasia.com/cna-insider/why-healthcare-workers-singapore-hospitals-resignations-2647746 (Accessed November 24, 2023).

[11] National Population and Talent Division. Population in Brief 2023. (2023). Available online at: https://www.population.gov.sg/files/media-centre/publications/population-in-brief-2023.pdf (Accessed February 7, 2024).

[12] Singapore Medical Association. Violence in the workplace - A survey on the experience of doctors in Singapore. Singapore Medical Association News. (2010). Available online at: https://news.sma.org.sg/4211/Violence.pdf (Accessed September 11, 2023).

[13] Ministry of Health Singapore. Annex findings and recommendations of the tripartite workgroup for the prevention of abuse and harassment of healthcare workers. (2022). Available online at: https://www.moh.gov.sg/docs/librariesprovider5/default-document-library/annex8e98f1b1026c44f7bc0416d0da5cbd2d.pdf (Accessed September 11, 2023).

[14] Ministry of Health Singapore. Launch of Tripartite Framework for the prevention of abuse and harassment In healthcare. Ministry of Health. (2023). Available online at: https://www.moh.gov.sg/newsroom/launch-of-tripartite-framework-for-the-prevention-of-abuse-and-harassment-in-healthcare (Accessed December 20, 2023).

[15] Recla-Vamenta G McKenna L McDonald E. Second-level nurses’ experiences of workplace violence: a scoping review. J Nurs Manag. (2023) 2023:1–24. doi: 10.1155/2023/6672952, PMID: 40225643 PMC11919165

[16] Arksey H O’Malley L. Scoping studies: towards a methodological framework. Int J Soc Res Methodol. (2005) 8:19–32. doi: 10.1080/1364557032000119616

[17] Tricco AC Lillie E Zarin W O’Brien KK Colquhoun H Levac D . PRISMA extension for scoping reviews (PRISMA-ScR): checklist and explanation. Ann Intern Med. (2018) 169:467–73. doi: 10.7326/M18-0850 30178033

[18] Tay GK Razak ARA Foong K Ng QX Arulanandam S. Self-reported incidence of verbal and physical violence against emergency medical services (EMS) personnel in Singapore. Australas Emerg Care. (2020) 24, 230–234. doi: 10.1016/j.auec.2020.09.001 32962931

[19] Tan Tock Seng Hospital News. More healthcare workers facing abuse - Tan tock Seng hospital (2021). Available online at: https://www.ttsh.com.sg/About-TTSH/TTSH-News/Pages/More-healthcare-workers-facing-abuse.aspx (Accessed September 12, 2023).

[20] Arnetz JE Hamblin L Ager J Luborsky M Upfal MJ Russell J . Underreporting of workplace violence. Workplace Health Saf. (2015) 63:200–10. doi: 10.1177/2165079915574684, PMID: 26002854 PMC5006066

[21] Erickson L Williams-Evans SA. Attitudes of emergency nurses regarding patient assaults. J Emerg Nurs. (2000) 26:210–5. doi: 10.1016/S0099-1767(00)90092-8, PMID: 10839847

[22] Alexander C Fraser J. Occupational violence in an Australian healthcare setting: implications for managers. J Healthcare Manag/American College Healthcare Executives. (2004) 49:377–90. doi: 10.1097/00115514-200411000-00007 15603114

[23] Åström S Karlsson S Sandvide Å Bucht G Eisemann M Norberg A . Staff’s experience of and the management of violent incidents in elderly care. Scand J Caring Sci. (2004) 18:410–6. doi: 10.1111/j.1471-6712.2004.00301.x, PMID: 15598249

[24] Nelson S Leslie K McCormick A Gonsalves J Baumann A Thiessen NJ . Workplace violence against nurses in Canada: a legal analysis. Policy Polit Nurs Pract. (2023) 24:239–54. doi: 10.1177/15271544231182583, PMID: 37403491 PMC10563371

[25] Wu S Zhu W Li H Lin S Chai W Wang X. Workplace violence and influencing factors among medical professionals in China. Am J Ind Med. (2012) 55:1000–8. doi: 10.1002/ajim.22097, PMID: 22886819

[26] Krishna A Soumyaja D Subramanian J Nimmi PM. The escalation process of workplace bullying: a scoping review. Aggress Violent Behav. (2023) 71:101840. doi: 10.1016/j.avb.2023.101840

[27] Minhat HS Sahiran MN. Malaysian family physician official journal of the academy of family physicians of Malaysia and family medicine specialist association of Malaysia. Malays Fam Physician. (2023) 18:61. doi: 10.51866/oa.312 38026573 PMC10664758

[28] Kim S Lynn MR Baernholdt M Kitzmiller R Jones CB. How does workplace violence–reporting culture affect workplace violence, nurse burnout, and patient safety? J Nurs Care Qual. (2023) 38:11–8. doi: 10.1097/ncq.0000000000000641 36409656

[29] Wirth T Peters C Nienhaus A Schablon A. Interventions for workplace violence prevention in emergency departments: a systematic review. Int J Environ Res Public Health. (2021) 18:8459. doi: 10.3390/ijerph18168459, PMID: 34444208 PMC8392011

[30] Somani R Muntaner C Hillan E Velonis AJ Smith P. A systematic review: effectiveness of interventions to de-escalate workplace violence against nurses in healthcare settings. Saf Health Work. (2021) 12:289–95. doi: 10.1016/j.shaw.2021.04.004 34527388 PMC8430427

[31] Aljohani B Burkholder J Tran QK Chen C Beisenova K Pourmand A. Workplace violence in the emergency department: a systematic review and meta-analysis. Public Health. (2021) 196:186–97. doi: 10.1016/j.puhe.2021.02.009, PMID: 34246105

[32] Berhane A Enquselassie F. Patient expectations and their satisfaction in the context of public hospitals. Patient Prefer Adherence. (2016) 10:1919–28. doi: 10.2147/PPA.S109982, PMID: 27703337 PMC5038575

[33] Barnett DJ Balicer RD Blodgett D Fews AL Parker CL Links JM. The application of the Haddon matrix to public health readiness and response planning. Environ Health Perspect. (2005) 113:561–6. doi: 10.1289/ehp.7491 15866764 PMC1257548

[34] Folkman S Moskowitz JT. Coping: pitfalls and promise. Annu Rev Psychol. (2004) 55:745–74. doi: 10.1146/annurev.psych.55.090902.141456 14744233

[35] Chan AOM Chan YH Kee JPC. Exposure to crises and resiliency of health care workers in Singapore. Occup Med. (2012) 63:141–4. doi: 10.1093/occmed/kqs202, PMID: 23223749

[36] Fletcher D Sarkar M. Mental fortitude training: an evidence-based approach to developing psychological resilience for sustained success. J Sport Psychol Action. (2016) 7:135–57. doi: 10.1080/21520704.2016.1255496

[37] Choi SY Kim H Park KH. Experience of violence and factors influencing response to violence among emergency nurses in South Korea: perspectives on stress-coping theory. J Emerg Nurs. (2021) 48, 74–84. doi: 10.1016/j.jen.2021.07.008 34538520

[38] Spelten E Thomas B O’Meara PF Maguire BJ FitzGerald D Begg SJ. Organisational interventions for preventing and minimising aggression directed towards healthcare workers by patients and patient advocates. Cochrane Database Syst Rev. (2020) 4. doi: 10.1002/14651858.cd012662.pub2 PMC7197696 32352565

[39] Rahman H. (2018). Abuse of Healthcare Workers. Singapore Medical Association News. Available online at: https://www.sma.org.sg/UploadedImg/files/Publications%20-%20SMA%20News/5005/Opinion.pdf (Accessed September 15, 2023).

[40] Assoc A Chua R. Workplace harassment in Singapore healthcare sector. (2025). Available online at: https://www.tal.sg/wshc/-/media/tal/wshc/resources/event-resources/presentation-slides/files/workplace-harrassment-in-singapore-healthcare-sector.ashx (Accessed February 14, 2025).

[41] Singapore Department of Statistics. Registered Health Personnel (End Of Period). Annual SINGSTAT [Internet]. Data.gov.sg. (2025). Available online at: https://data.gov.sg/datasets/d_bee760a2966a2e30c29b7eedb662b912/view?dataExplorerPage=18columnLegendPage=5 (Accessed June 2, 2025).

